# Evaluation of Serum CTRP-4 and CTRP-12 Levels in Hashimoto's Thyroiditis Patients: A Comparative Analysis with a Control Group and Their Correlation with Biochemical Factors 

**DOI:** 10.30699/ijp.2024.2025001.3276

**Published:** 2025-01-10

**Authors:** Doaa Jawad al-Husseini, Mohsen Saravani, Shahin Nosratzehi, Hamideh Akbari, Afsaneh Shafiei, Seyyed Mehdi Jafari

**Affiliations:** 1 *Metabolic Disorders Research Center, Golestan University of Medical Sciences, Gorgan, Iran*; 2 *Department of Biochemistry and Biophysics, Faculty of Medicine, Golestan University of Medical Sciences, Gorgan, Iran* *.*; 3 *Genetics of Non-communicable Disease Research Center, Zahedan University of Medical Sciences, Zahedan, Iran*; 4 *Department of Internal Medicine, School of Medicine, Zahedan University of Medical Sciences, Zahedan, Iran*; 5 *Clinical Research Development Unit, Sayad Shirazi Hospital, Golestan University of Medical Science, Gorgan, Iran*; 6 *Department of Clinical Biochemistry, School of Medicine, Iranshahr University of Medical Sciences, Iranshahr, Iran*

**Keywords:** Anti-TPO, Autoimmune diseases, CTRP-4, CTRP-12, biochemical Factors, Hashimoto Thyroiditis

## Abstract

**Background & Objective:**

Hashimoto's thyroiditis (HT) is one of the most common thyroid disorders and is characterized by manifestations attributed to thyroid gland damage and inflammatory conditions. Disturbances in thyroid hormones have physiological effects on lipoprotein metabolism and liver enzymes. CTRP family: C1q/TNF-related protein (CTRP) is an adipokine superfamily of proteins. Its essential role is anti-inflammatory activation, insulin sensitization, and regulation of blood lipids. In this study, we investigated the levels of CTRP-4 and CTRP-12 in the serum of HT patients and determined their association with biochemical factors.

**Methods:**

The study included 60 participants, divided into HT patients and control groups. Diagnostic criteria for HT patients included anti-thyroid peroxidase antibodies (anti-TPO)>50 IU/mL. Serum levels of CTRP-4, CTRP-12, and anti-TPO were measured using an Enzyme-Linked Immunosorbent Assay.

**Results:**

Our findings showed that the CTRP-4 and CTRP-12 levels in HT patients were higher than in the control groups (P=0.012 and P=0.003, respectively). HT patients also exhibited higher fasting blood glucose (FBG), cholesterol, TG, HDL, and LDL serum levels. Spearman's correlation analysis revealed a positive association between serum levels of CTRP-4 and CTRP-12 and anti-TPO (respectively, r= 0.295, *P*=0.022 and r = 0.346, *P*=0.007).

**Conclusion:**

Our findings showed that the CTRP-4 and CTRP-12 levels in HT patients were higher than those in the control groups. These factors probably play a role in the pathogenesis of Hashimoto's thyroiditis. The clinical significance of these factors should be evaluated in future studies.

## Introduction

Hashimoto's thyroiditis (HT), also known as chronic lymphocytic thyroiditis and chronic autoimmune thyroiditis, is an autoimmune disorder in which the immune system attacks thyroid gland cells, resulting in inflammation (1, 2). Diagnosis of HT is based on clinical characteristics and positivity for serum antibodies against thyroid peroxidase (TPO) and thyroglobulin (thyroid antigens) (3). HT is one of the most common thyroid diseases, and its incidence has increased over the last three decades (3). Approximately 5% of the global population is affected by Hashimoto's disease, with women being primarily affected (4, 5).

Adipokines are among the markers expressed in inflammatory diseases (6). The C1q/TNF-related protein (CTRP) family is a group of adipokines primarily secreted by adipose tissue. Fifteen members of this family (CTRP1–15) have been identified so far. They are involved in regulating various physiological and pathological processes and exhibit anti-inflammatory effects (7–10). C1Q/TNF-related protein 4 (CTRP-4) belongs to the CTRP superfamily and has been detected in humans, mice, and zebrafish (11). CTRP-4 is widely expressed in the brain, bone marrow stem cells, adipose tissue, and the circulatory system. It plays vital roles in metabolism, the immune system, and inflammatory responses (7, 11–13).

Complement-C1q/tumor necrosis factor-related protein-12 (CTRP-12; also known as adipolin) is a novel adipokine expressed in various tissues, especially subcutaneous adipose tissue (6, 14). It has multiple functions in both physiological and pathological processes, including regulating glucose and lipid metabolism, exerting anti-inflammatory effects, and modulating insulin sensitivity (6). The insulin-sensitizing activity of CTRP-12 is achieved by inhibiting gluconeogenesis and increasing glucose uptake in adipocytes and liver cells (15). In this context, a recent study found that serum CTRP-12 levels are lower in patients with type 2 diabetes (16).

Because CTRP-4 and CTRP-12 activities are involved in inflammatory processes, this study aims to compare the changes in serum levels of CTRP-4 and CTRP-12 in HT patients with those in a control group.

In the present study, we investigated the association between CTRP-4 and CTRP-12 levels and HT disease, as well as their relationships with various biochemical parameters, including triglycerides, high-density lipoprotein cholesterol (HDL-C), low-density lipoprotein (LDL) serum cholesterol, aspartate aminotransferase, and alanine aminotransferase.

## Material and Methods

After justification and written agreement, HT patients and healthy people were selected for cooperation. All the people enrolled in our study provided written and knowledgeable consent. Our study was approved by the Ethics Committee of the Golestan University of Medical Sciences (IR.GOUMS.REC.1401.154).

The total number of people in this research was 60 people, including 30 healthy people (control group) and 30 Hashimoto’s thyroiditis patients. The characteristics of the patients, including age, sex, weight, height, and duration of treatment, were determined. HT patients were included in this study based on the opinion of a specialist doctor, who was positive for anti-TPO antibodies, according to their medical history. Individuals without any history of systemic disease and negative for anti-TPO antibodies were included in this study as a control group.

### CTRP-4 and CTRP-12 Measurement:

After separating the serum, the serum CTRP-4 and CTRP-12 levels of the two groups (HT patients and control group) were determined using a human Enzyme-Linked Immunosorbent Assay (ELISA) kit (ZellBio GmbH, Germany). CTRP-4 and CTRP-12 proteins were added to wells pre-coated with anti-CTRP-4/12 monoclonal antibodies. Biotin-labeled anti-CTRP-4 and CTRP-12 antibodies were added to conjugate with streptavidin-HRP, forming an immunocomplex. After incubation and washing, unbound enzymes were removed, and then the substrate was added.

### Biochemical assays

The levels of Triglyceride, Total Cholesterol, High-density lipoprotein cholesterol (HDL-C), Aspartate aminotransferase (AST), and Alanine aminotransferase (ALT) were measured using automated analyzers using the Paadco kit (Iran). The Low-density lipoprotein cholesterol (LDL-C) level was calculated based on the Friedewald equation.

### Statistical analysis

The collected data were analyzed using SPSS 25 (SPSS Inc., Chicago, Ill., USA). The t-student statistical test was used to compare the average parameters of two groups for data with a normal distribution, and Mann-Whitney tests were used for data with a non-normal distribution. The results are reported as means ± standard deviations (SD).

## Results

### Description of the Studied Groups

In the present study, Hashimoto's thyroiditis patients were diagnosed according to high serum levels of anti-thyroid peroxidase antibodies (anti-TPO>50 IU/mL). All control subjects were negative for this test. Anti-TPO levels in Hashimoto's thyroiditis patients are significantly higher than in the control group (*P*<0.001).

Hashimoto's thyroiditis patients (30 people; 25 women and 5 men) and healthy controls (30 people; 25 women and 5 men). In the patient group, the average age of the participants was 42.35±10.85 years (range 21_65 years), and in the control group, the average age was 42.31±9.45 years (range 21_63 years). The mean BMI in patients with Hashimoto's thyroiditis was 24.42±2.96, and the mean BMI in healthy controls was 24.73±2.58 (*P*>0.05).

### Determination and Correlation of CTRP-4 and CTRP-12

CTRP-4 and CTRP-12 levels were compared between two groups of Hashimoto's thyroiditis patients and the control group. As shown in Table 1, CTRP-4 levels are significantly higher in HT thyroiditis patients than in the control group (Respectively 7.75±1.78 and 6.97 ± 1.91) (*P*<0.012). Also, CTRP-12 levels are significantly higher in Hashimoto's thyroiditis patients than in the control group (Respectively 242.62 ± 42.91 and 204.19 ± 62.75) (*P*<0.003).

The mean serum level of CTRP-4 was lower in females than in males (Respectively 7.24±1.89 and 7.89±1.80) (P=0.406). Also, the mean serum level of CTRP-12 in females was lower than that of males (Respectively 221.52±61.17 and 231.80±52.97) (*P*=0.879). 

### Correlation of CTRP-4 and CTRP-12 with Thyroid Hormones

The correlation of CTRPs and thyroid hormone profile in HT patients revealed a significant positive correlation between CTRP-4 and CTRP-12 (r = 0.851, *P*=0.000). There was a significant positive correlation between CTRP-4 and TSH (r = 0.261, *P*=0.44). There was a non-significant negative correlation between CTRP-4 and T3 and T4 levels. There was a significant correlation between CTRP-12 and TSH (r = 0.293, *P*=0.023). There was a non-significant relationship between CTRP-12 and T3 and T4 levels. The comparison of CTRPs with thyroid hormones is summarized in Table 2. 

### Determination and Comparison of Biochemical Variables in the Two Studied Groups

The biochemical variables evaluated between HT thyroiditis and control groups included fasting blood glucose (FBG), cholesterol, triglyceride, HDL, LDL, ALT, and AST (Table 3). Furthermore, the two groups also compared coronary risk index (Total cholesterol to HDL-C ratio) and Atherogenic index (LDL-C to HDL-C ratio). The serum levels of FBG, cholesterol, triglyceride, coronary risk index, and atherogenic index in Hashimoto's thyroiditis patients are significantly higher than in controls. The serum level of LDL and AST in Hashimoto's patients was non-significantly higher than in the control group. The serum level of HDL in Hashimoto's patients was significantly lower than in the control group. The serum level of ALT in Hashimoto's patients was non-significantly lower than in the control group.

### Correlation Between CTRPs and Biochemical Variables

The correlation between CTRPs and biochemical variables was investigated through Spearman's non-parametric test. As shown in Table 4, both CTRP-4 and CTRP-12 had a significant positive correlation with anti-TPO (respectively, r= 0.295, *P*= 0.022, and r = 0.346, *P*= 0.007). CTRP-4 and CTRP-12 values were also correlated (r = 0.851, *P*<0.001).

### Predictive value of CTRP-4 and CTRP-12 for Hashimoto's Thyroiditis

**Fig. 1:**
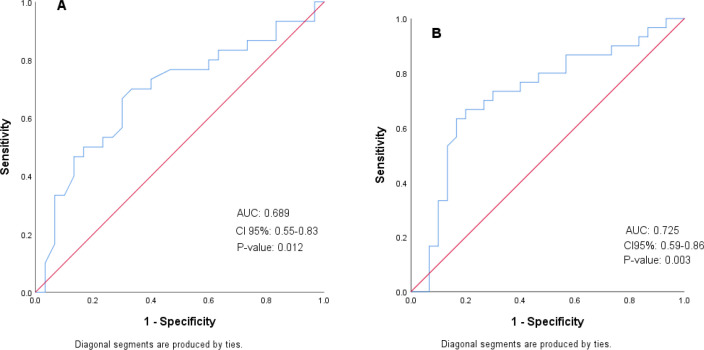
Receiver operating characteristic curves for Hashimoto's thyroiditis. A: CTRP-4. B: CTRP-12

The receiver operating characteristic curve was plotted for both CTRP-4 and CTRP-12. The area under the curve was obtained for each of them (Figure 1, respectively, A and B).

As seen in Figure 1, CTRP-12 had a larger area under the curve than CTRP-4. The optimal cut point with the highest sensitivity and specificity for each CTRP-4 and CTRP-12 is given in Table 5. As viewed in Table 5, CTRP-12 at the optimal cutoff point of 228.31 has better sensitivity, specificity, positive predictive value, and negative predictive value than CTRP-4 at the optimal cutoff point of 8.19 (ng/mL).

## Discussion

Hashimoto's thyroiditis (HT) is a chronic autoimmune disease and one of the main causes of hypothyroidism, leading to atrophy and fibrosis of the thyroid tissue, alongside the production of autoantibodies against thyroid cells and the destruction of thyroid follicles by infiltrated T cells (17). Patients with Hashimoto's often experience only mild symptoms during the early stages, which may take years to manifest. If left untreated for a prolonged period, Hashimoto’s thyroiditis can be associated with complications such as cardiovascular problems, high cholesterol, and anemia (18).

One group of compounds involved in inflammatory processes is the adipokine family. All CTRPs (CTRP1–15) share structural and sequence homology with the globular domain of adiponectin; each CTRP’s tissue of origin reflects its unique function in regulating glucose and lipid metabolism (19). Although no study has yet investigated the possible role of CTRP-4 and CTRP-12 in the pathogenesis of Hashimoto's thyroiditis, research on the impact of CTRPs in the development of autoimmune and inflammatory diseases is ongoing.

In this study, we examined serum levels of CRTP-4 and CTRP-12 in patients with Hashimoto's thyroiditis. Our findings showed that Hashimoto’s thyroiditis patients had significantly higher serum levels of CRTP-4 and CTRP-12 than healthy individuals.

An increase in CTRP-4 was also reported in herpes simplex encephalitis (an acute inflammatory disease), suggesting that higher CTRP-4 levels may contribute to the progression of this condition (20). Dai* et al.* found that increased CTRP-4 is linked with acute coronary syndrome (21). Nadimi Shahraki* et al.* showed that in coronary artery disease, which is inflammation-related, the serum level of CTRP-12 decreases compared to controls (6). Likewise, Fadai* et al.* reported a decrease in CTRP-12 levels in patients with coronary artery disease (22).

Hashimoto’s thyroiditis patients also displayed higher levels of FBG, cholesterol, triglycerides, HDL, and LDL. Hasret* et al.* observed that triglycerides, HDL, and LDL levels in Hashimoto’s patients are significantly higher than in controls (23). Similarly, Yetkin* et al.* reported increased levels of triglycerides, LDL, total cholesterol, and lipoproteins in Hashimoto’s patients (24). According to Azizi* et al.*, LDL, HDL, and total cholesterol levels in radioiodine-treated hypothyroid Graves’ patients on LT4 therapy are lower than in other groups (25). A study by Liu* et al.* demonstrated that individuals with autoimmune lupus nephritis had higher triglyceride and LDL levels than those with non-lupus nephritis (26). Another study indicated that LDL levels were lower in rheumatoid arthritis patients in the five years preceding disease onset than in healthy individuals (27). In a study by Vesna* et al.*, patients with Graves’ disease who received antioxidant supplementation in addition to methimazole had higher LDL levels than those treated only with methimazole (28). Serum LDL in psoriasis patients with diabetes was higher than in those with diabetes alone (29).

CTRP-4, containing the globular C1q bidomain, is notably secreted from the brain, adipose tissue, and bone tissue. CTRP-4 plays a major role in glucose and fat metabolism. In 2017, Lu Wang reported that, although excess CTRP-4 was associated with increased food intake in CTRP-4 transgenic mice, it significantly prevented hyperglycemia and obesity by reducing insulin resistance (13).

CTRP-12, another member of the CTRP family examined in this study, is notably secreted from adipose tissue, where its anti-diabetic properties are well-documented. Its circulating levels decrease in diabetes and obesity (30). In one study, Tan* et al.* showed that treating hepatocytes with CTRP-12 inhibited triglyceride synthesis. Moreover, in primary hepatocytes from mice, CTRP-12 suppressed triglyceride release by inhibiting cellular enzymes involved in VLDL production. These results suggest that upregulation of CTRP-12 expression correlates with reduced serum triglyceride levels during both fasting and postprandial states (31). In our study, although Hashimoto’s thyroiditis patients displayed higher serum CTRP-12 levels compared to controls, their serum levels of fasting blood glucose and lipid profile were also elevated. Hence, our results differ from this previous study regarding the relationship between CTRP-12 and triglyceride levels.

In another investigation, Sattar Gorgani* et al.* showed that CTRP-12 levels were significantly reduced in type 2 diabetic patients and that there was an inverse correlation between CTRP-12 concentrations and both FBG and HbA1c (16). Meanwhile, the present study found elevated levels of CTRP-12, FBG, total cholesterol, triglycerides, and HDL in Hashimoto’s thyroiditis patients, with no significant correlation between CTRP-12 levels and FBG.

Enomoto* et al.* demonstrated that production and secretion of CTRP-12 in adipose tissue and plasma were reduced in obese mouse models. They also showed that inflammation and endoplasmic reticulum stress in adipose cell lines led to decreased CTRP-12 expression (30).

Our results further revealed that anti-TPO levels in Hashimoto’s thyroiditis patients are significantly higher than in the control group (*P*<0.001). We observed a significant positive correlation between both CRTP-4 and CTRP-12 levels and anti-TPO, suggesting that increased CTRP-4 may be tied to pro-inflammatory conditions in Hashimoto’s thyroiditis. In another study, Yang Luo* et al.* found a positive and robust correlation between elevated CTRP-4 levels and reduced inflammation as well as inflammation-driven tumorigenesis in patients with ulcerative colitis (32).

**Table 1:** Comparison of CTRPs between the two studied groups

CTRPs	Hashimoto's thyroiditis	Control		*P*
	mean ± SD			
CTRP-4 (ng/mL)	7.75 ± 1.78		6.97 ± 1.91	0.012
CTRP-12 (pg/mL)	242.62 ± 42.91		204.19 ± 62.75	0.003

**Table 2:** Correlations of CTRPs with thyroid hormones

Correlations (Spearman’s)										
	CTRP-4	*P*	CTRP-12	*P*	TSH	*P*	T3	*P*	T4	*P*
TSH	0.261^*^	0.044	0.293^*^	0.023	1	.	-0.332^**^	0.010	-0.584^**^	0.000
T3	-0.092	0.487	0.000	0.998	-0.332^**^	0.010	1	.	0.383^**^	0.003
T4	-0.143	0.276	-0.073	0.577	-0.584^**^	0.000	0.383^**^	0.003	1.	.
CTRP-4	1	.	0.851^**^	0.000	0.261^*^	0.044	-0.092	0.487	-0.143	0.276
CTRP-12	0.851^**^	0.000	1	.	0.293^*^	0.023	0.000	0.998	-0.073	0.577

**. Correlation is significant at the 0.01 level (2-tailed).

*. Correlation is significant at the 0.01 level (2-tailed).

**Table 3:** Comparison of biochemical variables between the two studied groups

Biochemical variables	Mean ± SD		*P*
	Hashimoto's thyroiditis	Control	
FBG	105.67 ± 19.28	89.47 ± 12.47	0.001***
Cholesterol	170.57 ± 46.26	142.27 ± 24.82	0.005**
Triglyceride	118.27 ± 47.85	89.30 ± 31.94	0.013*
LDL	71.40 ± 21.22	64.83 ± 15.11	0.173
HDL	28.67 ± 9.86	36.57 ± 9.14	0.002**
AST	26.43 ± 20.47	21.27 ± 10.90	0.646
ALT	9.97 ± 4.87	11.17 ± 9.80	0.309
Coronary risk index	6.64 ± 3.40	4.13 ± 1.20	0.001***
Atherogenic index	2.67 ± 0.99	1.84 ± 0.47	0.001***

* *P*<0.05; ** *P*<0.01; *** *P*<0.001; FBG, fasting blood glucose; LDL-c, low-density lipoprotein; HDL-c, high density lipoprotein;

AST, Aspartate transaminase; ALT, Alanine transaminase

**Table 4:** Determining the correlation between CTRP4 and CTRP-12 with biochemical variables

Biochemical variables	CTRP-4		CTRP-12	
	R	*P*	R	*P*
FBG	0.193	0.139	0.240	0.065
Total cholesterol	0.116	0.378	0.057	0.664
Triglyceride	0.027	0.838	0.014	0.917
LDL-C	0.138	0.294	0.073	0.579
HDL-C	0.091	0.490	-0.063	0.630
AST	0.109	0.407	0.008	0.950
ALT	0.010	0.941	-0.079	0.548
Anti-TPO	0.295	0.022*	0.346	0.007**
Coronary risk index	0.012	0.928	0.068	0.604
Atherogenic index	0.111	0.397	0.131	0.318

* *P*<0.05; ** *P*<0.01; FBG, Fasting blood glucose; R, Correlation Coefficient; CTRP12, C1Q/TNF-related protein 12; CTRP4, C1Q/TNF-related protein 4; LDL-c, low-density lipoprotein; HDL-c, high density lipoprotein; AST, Aspartate transaminase; ALT, Alanine transaminase; anti-TPO, Antithyroid Peroxidase Antibody.

**Table 5:** The optimal cutoff point for CRP-4 and CRP-12 values to diagnose Hashimoto's thyroiditis

CTRPs	Maximum Youden index					
	Cut off	Sensitivity (%)	Specificity (%)	CI95%	PPV	NPV
CRTP-4 (ng/mL)	8.19	50	80	23.33-73.33	71.43	61.54
CRTP-12 (pg/mL)	228.31	66.67	80	26.67-86.67	76.92	70.59

CI, confidence interval; CTRP12, C1Q/TNF-related protein 12; CTRP4, C1Q/TNF-related protein 4; PPV, positive predictive value; NPV, negative predictive value.

## Conclusion

Our finding showed that both the CTRP-4 and CTRP-12 levels in HT patients were higher than the control groups and probably play a role in the pathogenesis of Hashimoto's thyroiditis. The clinical significance of these factors should be evaluated in future studies.
